# Regulatory network of genes associated with stimuli sensing, signal transduction and physiological transformation of appressorium in *Magnaporthe oryzae*

**DOI:** 10.1080/21501203.2018.1492981

**Published:** 2018-07-06

**Authors:** Wilfred Mabeche Anjago, Tengshen Zhou, Honghong Zhang, Mingyue Shi, Tao Yang, Huakun Zheng, Zonghua Wang

**Affiliations:** a Fujian University Key Laboratory for Plant-Microbe interaction, Fujian Agriculture and Forestry University, Fuzhou, China; b Plant Protection College, Fujian Agriculture and Forestry University, Fuzhou, China; c Institute of oceanography, Minjian University, FuzhouChina

**Keywords:** *Magnaporthe oryzae*, host sensing, appressorium development, sensing machinery, signal transduction

## Abstract

Rice blast caused by *Magnaporthe oryzae* is the most destructive disease affecting the rice production (*Oryza sativa*), with an average global loss of 10–30% per annum. Recent reports have indicated that the fungus also inflicts blast disease on wheat (*Triticum aestivum*) posing a serious threat to the wheat production. Due to its easily detected infectious process and manoeuvrable genetic manipulation, *M. oryzae* is considered a model organism for exploring the molecular mechanism underlying fungal pathogenicity during the pathogen–host interaction. *M. oryzae* utilises an infectious structure called appressorium to breach the host surface by generating high turgor pressure. The appressorium development is induced by physical and chemical cues which are coordinated by the highly conserved cAMP/PKA, MAPK and calcium signalling cascades. Genes involved in the appressorium development have been identified and well studied in *M. oryzae*, a summary of the working gene network linking stimuli sensing and physiological transformation of appressorium is needed. This review provides a comprehensive discussion regarding the regulatory networks underlying appressorium development with particular emphasis on sensing of appressorium inducing stimuli, signal transduction, transcriptional regulation and the corresponding developmental and physiological responses. We also discussed the crosstalk and interaction of various pathways during the appressorium development.

## Introduction

One of the most devastating diseases in rice is the rice blast, solely caused by the ascomycete fungus *Magnaporthe oryzae* (Ou 1985). Rice is a highly valuable cereal crop since half of the global population relies on it as their staple food. Food sustainability is the key challenge confronting the global community, considering the rapid growth of the world population (Ronald 2011). These concerns are further heightened due to enormous annual losses in rice yield caused by rice blast fungus (Ou 1985). Tremendous resources have been invested on studies aimed at gaining insights into molecular mechanisms employed by *M. oryzae* to successfully invade, colonise and destroy the host (Valent 1990). These efforts include the identification of valuable genetic targets for developing efficient and environment friendly anti-blast agents. Current blast disease management practices include introgression of dominant resistant genes (R-genes), application of fungicides and adaptation of proper cultural practices such as crop rotation (TeBeest et al. 2007).

*M. oryzae* is a haploid filamentous ascomycete that spreads via the production of asexual spores (Howard and Valent 1996; Talbot 2003). The conidia are borne sympodially on conidiophores (Howard 1994). The pyriform conidium contains three cells separated by septa. The life cycle commences with the conidium landing on host tissues followed by the germ tube elongation typically from the apical cell upon sensing the physical and chemical cues on host surface (Hamer et al. 1988). Studies have shown that the nucleus in the germinated cell migrates into the germ tube and undergoes a single round of mitosis to yield two daughter nuclei (Rossman et al. 1990; Howard and Barbara 1996). One of the nuclei moves into the germ tube and serves as the genetic source for the appressorium formation. Surface hydrophobicity and nutrient starvation are prerequisites for inducing the autophagic machinery in the conidium through which stored nutrients like glycogen and lipids are metabolised to empower the morphogenetic transformation during the appressorium development (Kershaw and Talbot 2009; Liu et al. 2016). The matured appressorium accumulates highly concentrated glycerol of ≈3.2 M, resulting in the generation of 8 MPa turgor pressure that creates physical force needed to breach the plant cuticle (Howard et al. 1991; De Jong et al. 1997). After gaining entry into host cells, *M. oryzae* invades in a hemibiotrophic lifestyle. Invasive hyphae are enclosed by the host extra-invasive-hyphal membrane within 36 hours of biotrophic growth and extend into the neighbouring cells via the plasmodesmata to cause the necrosis of host tissues (Couch et al. 2005; Kankanala et al. 2007; Nishizawa et al. 2016). Although the genetic determinants facilitating this hemi-biotrophic transition are mostly unknown, there are suggestions that effector proteins secreted by *M. oryzae* play a prominent role in suppressing host immunity during this transition (Zhang et al. 2014b). A recent study has shown that secreted enzymes with hydrolytic activities such as cellulases and xylanases and toxins with cell death-inducing activities are deployed to facilitate necrotic infection (Kankanala et al. 2007). The disease symptoms include necrotic lesions formed on either leaves, nodes, neck or panicles of the host plant, as *M. oryzae* is capable of infecting a wide range of tissues (Skamnioti and Sarah 2009). Conidia formed from lesions are scattered to neighbouring plants by wind or splash to initiate the subsequent infection, resulting in disease advancement (Heath et al. 1990).

**Figure 1.**
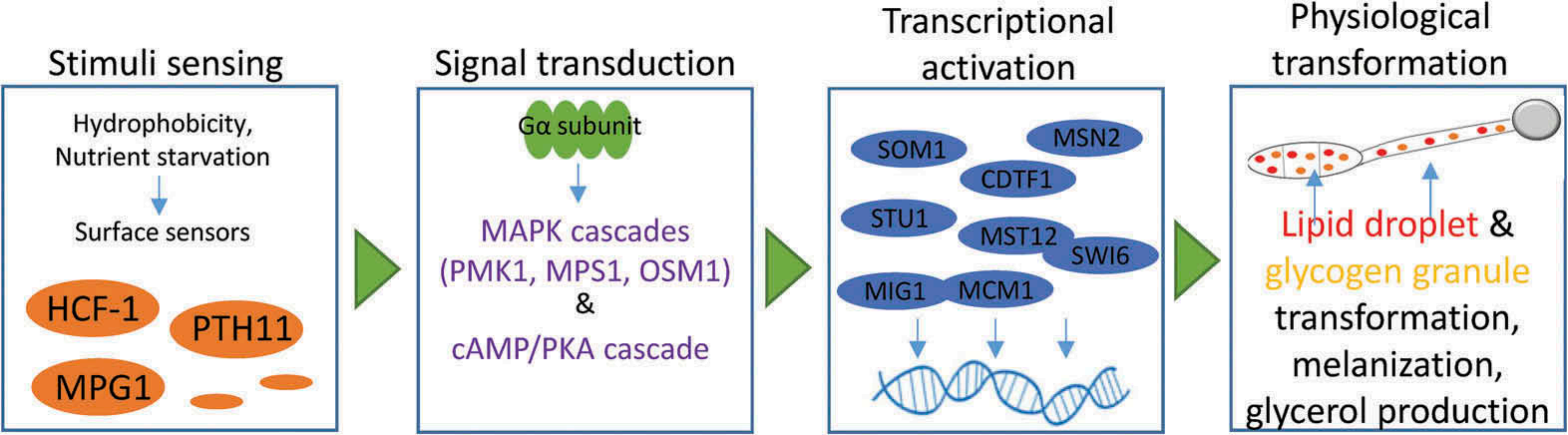
Control process of the gene regulatory network for appressorium development and maturation in *M. oryzae*. Schematic of the appressorium development process starting from environmental stimuli sensing to signal detection and transduction transcription activation and the resulting metabolic flow transformation for cell wall melanization and glycerol production prior to penetration.

Provided the essential role of appressorium development in *M. oryzae* pathogenesis, it is necessary to reconsider the functions of those genes within a broad context of pathway crosstalk and gene network (Figure 1). Therefore, the following discussion will focus on addressing the need to provide a network model for genes directly associated with stimuli sensing, signal transduction and physiological transformation of appressorium in *M. oryzae*.

## The role of hydrophobins in appressorium morphogenesis

Hydrophobins are about ≤20 kDa small secreted proteins that form rodlet layer on the spore surface and hydrophobin monomer on aerial hyphae of filamentous fungi (Bayry et al. 2012). The hydrophobic, water-resistant rodlet layer plays an essential role in facilitating wind dispersal of spores produced by *Aspergillus nidulans* (Grünbacher et al. 2014) and also shield spores from being recognised by immune cells (Bruns et al. 2010). Six hydrophobin genes, *DEW*-A, *DEW*-B, *DEW*-C, *DEW*-D, *DEW*-E and *ROD*-A, maintain spore hydrophobicity via formation of spore rodlet layer in *A. nidulans* (Grünbacher et al. 2014). The disruption of *ROD*-A undermined the efficient spore dispersion due to lack of rodlet layer (Stringer et al. 1991). Hydrophobins self-assemble into amphipathic monomers in an aqueous or non-aqueous environment to minimise surface tension of aerial hyphae in water–air interface (Wessels 1996; Kwan et al. 2006). These proteins additionally contribute to surface hydrophobicity of aerial hyphae and protect the aerial hyphae against waterlogging (Wessels et al. 1991). Aerial hyphae with compromised surface hydrophobicity are highly wettable. For instance, the deletion of *MHP*1 and *MPG*1 in *M. oryzae* triggered the formation of highly wettable hyphae in the respective mutant strains (Talbot et al. 1993; Kim et al. 2005); a similar phenotype was observed in *Fusarium graminearum* ∆*hyd5* strains (Minenko et al. 2014), and *Trichoderma reesei* ∆*trhfb3* strains (He et al. 2017). However, the deletion of three hydrophobin encoding genes *BHP*1, *BHP*2 and *BHP*3 in *Botrytis cinerea* did not alter the surface hydrophobicity of conidia and hyphae produced by the respective gene deletion mutants (Terhem and Van Kan 2014). Also, the deletion of hydrophobin genes (*HYD*1, *HYD*2 and *HYD*3) in *Clonostachys rosea* only attenuated the hydrophobicity of the aerial hyphae but not conidial hydrophobicity (Dubey et al. 2014). These observations suggest that the role of hydrophobins in regulating surface hydrophobicity is not conserved in filamentous fungi. This might be due to different infection strategies and mechanisms employed by these pathogens to invade the host or existence of other unidentified hydrophobin proteins playing redundant roles in regulating surface hydrophobicity. Hydrophobins are categorised into two main classes: Class I and Class II based on their solubility property, hydropathy characteristic and structure formed in the hydrophobic–hydrophilic interface (Wessels 1996).

Numerous research findings have shown that hydrophobins are crucial for pathogenicity in phytopathogens. For instance, *MPG*1 and *MHP*1 essentially promote sporulation and full virulence of *M. oryzae* (Talbot et al. 1993; Kim et al. 2005). Northern blot analysis showed a higher induction of *MPG*1 during early (12 hpi) and late (96 hpi) stages of plant infection (Talbot et al. 1993); however, MHP1 only showed enhanced expression activities at late stages (96 and 120 hpi of infection) (Kim et al. 2005).

Recent research demonstrated that *MPG*1 assembles into functional amyloid structures through surface driven mechanism while *MHP*1 forms non-fibrillary structures on hydrophobic–hydrophilic interface (Pham et al. 2016). The *MPG*1, *MHP*1 and Cutinase2 (*CUT*2) interaction highlights the existence of an intricate relationship between hydrophobins and cutinases during appressorium formation and host penetration in rice blast causing agent. These findings underscore the central roles of hydrophobins proteins in promoting the conidial and mycelial hydrophobicity and appressorium morphogenesis in response to extracellular signals in *M. oryzae*.

## The impact of chitosan and chitin-binding proteins on hydrophobicity sensing and appressorium initiation

Controversy over the role of chitosan in germling adhesion, appressorium morphogenesis and fungal pathogenesis has been cleared after the characterisation of chitosan synthesising enzymes, chitin deacetylates (CDAs) in *M. oryzae* (Geoghegan and Gurr 2016). The disruption of CDAs compromised germling ability to successfully adhere and form appressorium on an artificial hydrophobic surface (Geoghegan and Gurr 2016). However, CDAs deletion mutants were pathogenic on susceptible host suggesting a specialised role of CDA gene family in signal perception and germling adhesion during appressorium initiation (Geoghegan and Gurr 2016). In *Cryptococcus neoformans* CDAs (Cda1, Cda2 and Cda3) maintain cell wall integrity, regulate bud separation and promote stress tolerance (Baker et al. 2007). Chitosan is a byproduct of chitin deacetylation and a polymer of β-1,4-glucosamine that accumulates in the germ tube and appressorium of *M. oryzae* (Geoghegan and Gurr 2016) and in spore cell wall of root endophyte, *Pochonia chylamydosporia* (Escudero et al. 2016). In nematophagous fungi, *P. chylamydosporia* chitosan enhances appressorium formation, proteolytic activity and fungal infectivity on nematode eggs (Escudero et al. 2016).

The novel Cbp1-chitin binding protein mediates the timely perception of appropriate environmental cues which is a vital prerequisite for appressorium differentiation in *M. oryzae* (Kamakura et al. 2002). Cbp1 is secreted into the extracellular environment during germ tube development to facilitate the cell adhesion and initiation of appressorium contact point on hydrophobic surface (Kamakura et al. 2002; Nakajima et al. 2010). Targeted gene replacement of CBP1 altered appressorium differentiation on an artificial hydrophobic surface and resulted in the formation of abnormally long germ tubes due to defects in hydrophobicity sensing (Kamakura et al. 2002). Recent findings have shown that CBP1 plays overlapping role with Msb2 in activating the PMK1 pathway during appressorium differentiation; however, the existence of a physical interaction between these two proteins is yet to be proven experimentally (Wang et al. 2015).

## The role of cutin and cuticular wax in appressorium morphogenesis

The plant cuticle is composed of cutin and cuticular waxes covering all the aerial parts of plants (Kunst and Samuels 2009). Cutin forms the core structural polymer of C16 and C18 hydroxy and epoxy medium chain fatty acids while the cuticular wax comprises of a mixture of very long chains fatty acid derivatives as well as alkaloids such as triterpenoids and phenypropanoids (Jetter et al. 2007). Plant cuticle serves as a protective barrier against invading microbes and insects and prevents excessive water loss and desiccation (Kunst and Samuels 2009). The topography, hardness and hydrophobic nature of the plant cuticle are essential chemical and physical cues that trigger appressorium formation in *M. oryzae* (Gilbert et al. 1996). These cues have also been reported to elicit appressorium formation in other foliar pathogens such as *Ustilago myadis* (Mendoza‐Mendoza et al. 2009), and *Colletotricum* spp. (Dickman et al. 2003). Notably exogenous addition of selected cutin monomers (*cis*-9, 10-epoxy-18-hydroxyoctadecanoic acid and *cis*-9-octadecen-1-ol) and wax (1,16-hexadecanediol and 1,16-hexadecanedial) in nanomolar range induced appressorium formation on a host and non-host plants implying appressorium induction is not specific to host plants (Gilbert et al. 1996). The detail mechanisms underlying specificity of Msb2-mediated cutin monomer and surface hydrophobicity sensing, as well as the exact role of Sho1 in leaf wax sensing, are still unknown. It was, however, suggested that these proteins might play overlapping role in sensing appressorium inducing signals (Liu et al. 2011). Surprisingly, targeted gene deletion of *M. oryzae MoMSB*2 and *MoSHO*2 did not significantly alter the progression of appressorium differentiation on host surface (Liu et al. 2011) From these observations, the likely existence of yet to be identified proteins in the rice blast fungus with an overlapping role in mediating appressorium morphogenesis was speculated. This hypothesis was confirmed recently after the CFEM (conserved fungal-specific extracellular membrane spanning) domain of *PTH11* like GPCR (G-protein coupled receptors) was identified and characterised in *M. oryzae*. Water wettability, Infection, Surface sensing and Hyper-conidiation (*WISH* GPCR) functions as an upstream effector for sensing diverse extracellular stimuli during appressorium differentiation in *M. oryzae* (Sabnam and Barman 2017).

Parasitic pathogens also secrete cutinases, cell wall degrading enzymes, during formation of infection structures. Cutinases facilitate spore adhesion in *Colletotrichum graminicola* (Pascholati et al. 1993), *Uromyces viciae-fabae* (Deising et al. 1992) and *Blumeria graminis* (Pascholati et al. 1992), carbon acquisition in *Venturia inequalis* (Köller et al. 1991) and also promote the virulence of *M. oryzae* (Skamnioti and Gurr 2007). Cutinase-mediated degradation of plant cuticle into cutin monomers elicits the cAMP/PKA and DAG/PKC signalling cascades and triggers appressorium formation in *M. oryzae* (Skamnioti and Gurr 2007; Liu et al. 2011). The *M. oryzae* genome encodes 17 putative cutinases (Dean et al. 2005) including Cutinase1 (*CUT*1) and Cutinase2 (*CUT*2) that have been previously characterised (Sweigard et al. 1992; Skamnioti and Gurr 2007). The expression of Cut2 significantly upregulated during appressorium maturation and host penetration underscores its role in promoting appressorium formation, host penetration and virulence of *M. oryzae* (Skamnioti and Gurr 2007) and justifies the need to experimentally evaluate role of remaining members of the cutinase family of proteins in appressorium formation and infectious development of *M. oryzae* and other pathogenic fungal species.

## Signal transducing cascades associated with appressorium formation

The central role of MAPK pathways is to transmit and integrate signals from a broad range of stimuli to target proteins that produce physiological response (Pearson et al. 2001). They regulate numerous biological processes like apoptosis, cell differentiation, stress response and cell proliferation in mammalian systems (Keyse 2000; Pathak et al. 2013). The MAPK pathway comprises three highly conserved protein kinases including MEKK or MAPKKK (Mitogen-Activated Protein Kinase Kinase Kinase), MEK or MAPKK (Mitogen-activated Protein kinase Kinase) and MAPK (Mitogen-Activated Protein Kinase). The pathway activation proceeds through a series of phosphorylation events in the MEKK–MEK–MAPK cascade (Robinson and Cobb 1997). The phosphorylated MAPK migrates to the nucleus and phosphorylates the downstream effectors including transcriptional factors, enzymes and protein kinases for signal amplification and mediation of a wide array of biological processes (Keyse 2000).

The highly conserved MAPK pathway plays a crucial role in regulating appressorium formation, and appressorium penetration in *M. oryzae*. Three MAPKs proteins including Pmk1, Osm1 and Mps1 directly modulate different signalling pathways associated with appressorium development, invasive growth and osmotic stress in *M. oryzae* (Xu and Hamer 1996; Xu et al. 1998; Dixon et al. 1999). The evolutionary conserved Pmk1 MAPK homologous to the *Saccharomyces cerevisiae* Fuss/kss1 regulates pheromone response pathway (Xu and Hamer 1996) and invasive growth, appressorium formation and pathogenicity in *M. oryzae* (Errede et al. 1993).

Besides Pmk1, additional two upstream kinases MEKK-Mst11 and MEK-Mst7 identified have been shown to play a crucial role in promoting appressorium formation (Park et al. 2006). The 12–20 AA MAPK docking site located at the N-terminus of Mst-7 functions in stabilising the interaction between Pmk1 and Mst7 during signal transduction. Mutation of this docking site abolished Mst7-Pmk1 interaction blocking appressorium formation although the kinase activity of Mst7 was still retained (Zhao and Xu 2007). An adaptor protein *MST*50 mediates the Mst11 and Mst7 association in *M. oryzae*. Recent interaction analysis of Mst50 protein revealed that the sterile alpha domain exclusively interacts with an upstream protein kinase Mck1 associated with the MPS1 pathway under cell wall and cell membrane-directed oxidative stress (Li et al. 2017a). More so, the Mst50-Histidine kinase (Hik1) interaction promotes the phosphorylation of Osm1 under similar oxidative stress conditions (Li et al. 2017a). This confirms the existence of pathway crosstalk among the components of MAPK signalling cascade during fungal development and stress response.

The Mps1-MAPK crucially regulate the development of functional and successful penetration of host tissues by *M. oryzae* (Xu et al. 1998), it also promotes the early phase of appressorium differentiation in *Colletotrichum lagenarium* (Kojima et al. 2002), and colony pigmentation in *Colletotrichum heterostrophus* (Eliahu et al. 2007). The Osm1-MAPK regulates the osmotic stress response pathway in filamentous fungi (Dixon et al. 1999). However, it is important to note that the OSM1 gene deletion mutant plays a dispensable role in glycerol accumulation, appressorium turgor generation and pathogenicity, although its *S. cerevisiae* homolog Hog1 regulates cellular turgor in response to high osmolarity (Brewster et al. 1993).

The cyclic AMP (cAMP) is another well-studied signalling pathway that transfers the extracellular stimuli required for the appressorium morphogenesis in *M. oryzae* (Bahn et al. 2007). The cAMP pathway plays a central role in regulating cellular proliferation and metabolism in budding yeast (Talbot 2003). Upstream signal transducers that exert a regulatory influence on cAMP synthesis during the infection-related morphogenesis comprise a heterotrimeric G-proteins complex of Gα subunit (Mag A, Mag B and Mag C) (Li et al. 2007), Gβ subunit (Mgb1 and Mgb2) and Gϒ subunit (Magg1) (Nishimura et al. 2003). Regulator of G-protein signalling (RGS) negatively regulates all Gα Subunits in *M. oryzae* (Liu et al. 2007). Also, excessive accumulation of intracellular cAMP was detected in the RGS gene deletion mutant and in the point mutant of magA^G187S^ (RGS insensitive Gαs) or magA^Q208L^ (without GTPase activity) that generated appressorium on a hydrophilic surface (Liu et al. 2007). Upon detection of extracellular stimuli like surface hydrophobicity, Gα subunit dissociates from the G-proteins complex and binds to the membrane-bound Adenylate cyclase (*MAC*1) which in turn is activated and converts ATP to cAMP (Adachi and Hamer 1998; Deka et al. 2016). One of the most well-known cell-surface membrane proteins that integrate the hydrophobic signal is *PTH*11, whose gene deletion mutant was non-pathogenic to the host due to a defect in appressorium differentiation (DeZwaan et al. 1999). Research findings currently available showed that the CFEM (Conserved fungal-specific extracellular membrane spanning) domain of Pth11 is vital for redox homeostasis, appressorium formation and virulence in *M. oryzae* (Kou et al. 2017).

Generation of an intracellular cAMP signal is essential for successful initiation of appressorium differentiation on an inductive surface (Adachi and Hamer 1998). The cAMP/PKA signal cascade is further mediated via its binding with the PKA regulatory subunit *SUM*1, allowing the release of the PKA catalytic subunit for phosphorylating the downstream targets in the nucleus like transcriptional factors including *CDTF*1, *SOM*1, *MSTU*1 in *M. oryzae* (Filippi 2004) and *SFL*1 in budding yeast (Song and Carlson 1998; Galeote et al. 2007). The two catalytic subunits CpkA and Cpk2 play specific and overlapping functions in regulating infection-related morphogenesis and pathogenicity of *M. oryzae* (Li et al. 2017b). Recent findings show that PKA activity is essential for appressorium formation and phosphorylation of SFL1 in *M. oryzae* (Li et al. 2017b). The activation of Sfl1 relieves Cy8-Tup1 which is a co-repressor that suppresses genes required for hyphal growth and appressorium development in *M. oryzae* (Li et al. 2017b).

Calcium signalling cascade is another crucial pathway shown to regulate appressorium development and pathogenicity in *M. oryzae* (Lee and Lee 1998) and *Colletotricum* spp. (Kim et al. 1998; Sakaguchi et al. 2008). The cytosolic levels of intracellular calcium are maintained at optimum levels (50–100 nM) by calcium pumps and transporters to prevent activation of calcium-dependent apoptosis in cells (Zelter et al. 2004). The calcium-dependent pathway regulates numerous cellular processes involving cell cycle, metabolism, and polarity in higher organisms (Berridge 1993). The activation of the calcium signalling cascade involves the interaction of ligands (growth factors and enzymes) with membrane surface receptors comprising heterotrimeric G proteins in the eukaryotic system (Nishizuka 1995). This association causes β-subunit dissociation from the complex and activation of membrane-bound protein phospholipase C. The rice blast genome encodes five phospholipase enzymes (*PLC*1, *PLC*2, *PLC*3, *PLC*4 and *PLC*5) that regulate conidiation, appressorium development and pathogenicity in *M. oryzae* (Choi et al. 2011). Besides, *PLC*1 also modulates calcium flux in *M. oryzae* and mammals (Rho et al. 2009). The activated phospholipase C hydrolyses PIP2 (phosphatidlyl inositol-4,5-bisphosphate) to DAG (Diacylglycerol) and IP3 (inositol-1,4,5-triphosphate). The released secondary messenger (DAG) activates protein kinase C(PKC) while IP3 triggers the release of Ca^2+^ from storage and binds to calcium-dependent targets comprising calcium/calmodulin (CaM)-dependent protein phosphatase, calcineurin (Buchner 1995). These calcium-dependent proteins and PKC traverse the nucleus to activate downstream effectors and transcriptional factors through phosphorylation (Dean 1997). In *M. oryzae*, a calcineurin-dependent transcription factor, Mo*CRZ*1, is required for normal vegetative growth, appressorium function and pathogenicity (Choi et al. 2009). The calcineurin is a heterodimeric protein consisting of a catalytic subunit (calcineurin A) and a regulatory subunit (calcineurin B). The catalytic binding subunit, *MCN*A, regulates infection-related morphogenesis and pathogenicity in *M. oryzae* (Choi et al. 2009). Previous findings have highlighted the existence of pathway crosstalk between calcium/calcineurin and MAPK signalling pathway during cell fusion step of mating in *S. cerevisiae* (Muller et al. 2003).

## Transcriptional factors regulating appressorium formation and pathogenicity

Transcriptional factors play crucial roles in activating gene expression in response to extracellular stimuli. Ample research demonstrations have shown that many of the transcriptional factors identified also play diverse roles that facilitate the progression of normal appressorium morphogenesis and pathogenicity of *M. oryzae* (Kim et al. 2009; Soanes et al. 2012). For instance, *MoSOM*1 which is a transcription factor serves as a phosphorylation target for CpkA along the cAMP/PKA pathway in *M. oryzae*. The physical interaction between MoSom1 and CpkA occurs in the presence of cAMP (Yan et al. 2011). MoSom1 also interacts with two other transcription factors namely MoCdtf1 and MoStu1 to regulate appressorium initiation and turgor generation and pathogenesis of *M. oryzae*. More so, MoCDTF1, MoSOM1 and MoMSB2 are also involved in the regulation of sporulation and development of functional appressorium in *M. oryzae* (Yan et al. 2011). Subsequently, ∆*mosom*1 and ∆*mocdtf*1 strains were non-pathogenic and failed to induce lesions on both susceptible rice and barley seedlings (Yan et al. 2011). Mst12 represents one of the well-studied transcription factors which is phosphorylated by the Pmk1-MAPK (Hamel et al. 2012). As a result, the disruption of *MoMST*12 in *M. oryzae* triggered the formation of non-functional appressorium in the ∆*mst*12 strains. The appressorium formed by the defective strains also failed to mobilise the turgor required to physically breach host cuticle for successful invasion (Park et al. 2002; Zhou et al. 2011). Additional reports showed that the MoMcm1 protein which serves as a downstream target for Pmk1-MAPK equally plays a crucial role in promoting the development of functional appressorium and host penetration. Further research demonstrations have shown that MoMcm1 and MoMst12 assume overlapping functions in regulating cell polarity and turgor generation in appressorium (Park et al. 2002; Zhou et al. 2011). The deletion of *MoMCM*1 phenotyping the mst12 mutant produced appressoria unable to penetrate the plant surface effectively. The two downstream targets of Mps1 MAPK cascade are Mig1 and Swi6 essential in regulating appressorium morphogenesis and cell wall integrity (Shiozaki and Russell 1996; Mehrabi et al. 2008; Qi et al. 2012). Targeted gene deletion of *MoSW*I6 compromised the cell wall integrity and suppressed the expression of extracellular enzymes implicated in the maintenance of cell wall rigidity during host penetration. These results revealed that the Mps1-MAPK pathway plays a regulatory role in promoting fungal transcriptomic reprogramming events necessary for successful host penetration and suppression of host defence response. Furthermore, Zhang et al. in their affinity purification studies identified a putative zinc finger transcription activator MoMsn2 and proceeded further to show that MoMsn2 plays an essential role in the appressorium development and enhance the pathogenicity of *M. oryzae* (Zhang et al. 2014a). MoMsn2 interacted with MoOsm1 *in vivo* suggesting that MoMsn2 regulates some downstream genes in stress response (Zhang et al. 2014a). A homeobox transcription factor Hox7 was found to be essential in appressorium formation and pathogenicity in *M. oryzae*. Addition of appressorium inducing chemicals such as cAMP and 1, 16-hexadecanediol could not restore the appressorium formation defect in Mohox7 mutant on hydrophobic and hydrophilic surfaces suggesting *HOX*7 is a crucial regulator for appressorium morphogenesis in *M. oryzae* (Kim et al. 2009).

## The essential role of glycerol production in appressorium function

During appressorium maturation, osmolytes like glycerol are accumulated intracellularly to a concentration of about 3.2 M and used in generating turgor pressure to break down plant cuticle (De Jong et al. 1997). However, genetic mechanisms underlying the physiological transformation for turgor generation are still unknown. Storage carbohydrates such as glycogen and trehalose are mobilised and degraded during the conidial germination to fuel the appressorium development (Dixon et al. 1999). At present, there is no direct experimental evidence linking turgor generation and glycogen metabolism, since glycogen is quickly degraded in the early appressorium (Thines et al. 2000a). The catabolism of these carbohydrates may provide the intermediate metabolites into the glycerol production pathway. Diverse cellular pathways contribute to glycerol production in *M. oryzae* via utilising dihydroxyacetone phosphate, dihydroxyacetone, glyceraldehyde or triacylglycerol as precursors (Thines et al. 2000b). Dihydroxyacetone, a glycolytic intermediate, is reduced to glycerol-3-phosphate by an NAD+-dependent glycerol phosphate dehydrogenase (*GPD*). Glycerol-3-phosphate is further dephosphorylated to glycerol by glycerol-3-phosphatase, which includes two homologs encoded by *HOR*1 and *HOR*2 in *S. cerevisiae* (Hirayama et al. 1995, Norbeck et al. 1996). Gene replacement of the two homologous genes namely *GPD*1 and *GPD*2 resulted in mutants with a decreased accumulation of intracellular glycerol and increased sensitivity to osmotic stress as compared to wild-type in *S. cerevisiae* (Albertyn et al. 1994). However, activities of *GPD* could not be detected during appressorium maturation, raising the possibility of an alternative glycerol production route needed to build up turgor pressure for appressorium penetration (Thines et al. 2000a).

Enzymatic analysis showed triacylglycerol lipases were active during appressorium development and remained high throughout the maturation process until the generation of turgor pressure (Thines et al. 2000a). However, disruption of the triacylglycerol lipases encoding genes *TGL1-1, TGL1-2, TGL2, TGL3-1, TGL3-2, HDL1, HDL3* and *VTL1* caused no significant effect on virulence and appressorium function in *M. oryzae*. This may be due to the presence of unidentified lipases contributing to intracellular lipolysis during appressorium development (Wang et al. 2007). Fatty acids liberated from the glycerol moiety are degraded via Beta oxidation to yield acetyl-CoA and channelled into the citric acid cycle for energy supply. Mutations of peroxisomal fatty acids β-oxidation encoding genes MFP1 and PEX6 delayed the mobilisation and degradation of the lipid droplets in the appressorium and abolished plant infection and penetration (Wang et al. 2007). Non-functional and less pigmented appressoria were formed by *pex1* mutants in *M. oryzae* (Deng et al. 2016). Cytological studies unveiled lipid bodies are mobilised from the germinating conidium and degraded during the appressorium maturation and turgor generation (Weber et al. 2001). This metabolic process is regulated by cAMP/PKA and Pmk1 MAPK signalling pathways, as mutations of genes controlling these pathways caused defects in appressorium morphogenesis, decreased glycerol production and subsequent loss of turgor generation (Xu and Hamer 1996; Xu et al. 1997). Lipid mobilisation and degradation were slowed down in PMK1 deletion mutant compared to wild type (Xu and Hamer 1996), as lipid droplets were retarded in the conidium after 48-h induction, finally causing failed appressorium differentiation. On the other hand, the deletion mutant of the PKA catalytic subunit encoding gene CpkA displayed a 50% decrease in the triacylglycerol lipase activity during appressorium maturation compared to wild type (Xu et al. 1997). Though lipid translocation from conidia to appressoria was unaffected, lipolysis did not take place during maturation process in the CpkA mutant (Mitchell and Dean 1995). The ∆*sum*1-99 mutant possessing a mutation in the regulatory subunit of PKA exhibited a different phenotype in lipid mobilisation and utilisation during the appressorium development, as the process took place within 12 h of induction (Adachi and Hamer 1998).

These observations underscored the critical role of PKA catalytic subunit as functionally antagonistic to the regulatory subunit in lipid catabolism during glycerol production and turgor generation.

A peroxisomal carnitine acetyltransferase (CAT) activity encoded by *PTH*2 in *M. oryzae* essentially mediates the efficient mobilisation of lipids to the appressorium. Deletion of *PTH*2 impaired the appressorium function and pathogenicity of *Mopth2* strains on a susceptible host plant. CAT catalysed the conversion of acetyl COA to acetylcarnitine and transported across intracellular membranes (Bhambra et al. 2006). *MoCRC*1 gene encoding a carnitine-acylcarnitine carrier protein is also involved in the normal appressorium functioning during turgor generation and penetration. The *MOCRC*1 mutants were nonpathogenic due to malfunctioned appressorium (Yang et al. 2012).

## Conclusion

Though well studied, appressorium development is a complicated morphogenetic process that is finely coordinated by an array of genes and signalling pathways that ensures the successful host penetration. Understanding the regulatory network underlying the appressorium development from aspects of stimuli sensing, signal transduction, transcriptional regulation to physiological response lays out the foundation for combating the rice blast disease via biological and chemical means. In this review, we summarised the genes that constitute the regulatory network from extracellular signal detection and transduction to nuclear transcriptional activation and the resulting physiological transformation during the appressorium development and infection on rice in *M. oryzae*. With the advancement of in-depth sequencing and metabolomics profiling technologies, aggregating efforts will be made to more accurately delineate the transcriptional rewiring and metabolic maps underpinning the appressorium development in *M. oryzae*.
