# Erratum

**DOI:** 10.1080/21501203.2018.1509563

**Published:** 2018-08-23

Anjago, W.B., Zhou, T., Zhang, H., Shi, M., Yang, T., Zheng, H., Wang, Z. 2018. Regulatory network of genes associated with stimuli sensing, signal transduction and physiological transformation of appressorium in *Magnaporthe oryzae*. Mycology. https://doi.org/10.1080/21501203.2018.1492981

When the above article was first published online, the asterisk symbol for the footnote ‘*These authors contributed equally to this work.’ was not included for the author Wilfred Mabeche Anjago.

This has now been corrected in the online and print versions.

Taylor and Francis apologises for this error.

